# Designing process evaluations using case study to explore the context of complex interventions evaluated in trials

**DOI:** 10.1186/s13063-020-04880-4

**Published:** 2020-11-27

**Authors:** Aileen Grant, Carol Bugge, Mary Wells

**Affiliations:** 1 School of Nursing, Midwifery and Paramedic Practice, Robert Gordon University, Garthdee Road, Aberdeen, AB10 7QB UK; 2 Faculty of Health Sciences and Sport, University of Stirling, Pathfoot Building, Stirling, FK9 4LA UK; 3 Department of Surgery and Cancer, Imperial College London, Charing Cross Campus, London, W6 8RP UK

**Keywords:** Process evaluation, Context, Trials, Case study design

## Abstract

**Background:**

Process evaluations are an important component of an effectiveness evaluation as they focus on understanding the relationship between interventions and context to explain how and why interventions work or fail, and whether they can be transferred to other settings and populations. However, historically, context has not been sufficiently explored and reported resulting in the poor uptake of trial results. Therefore, suitable methodologies are needed to guide the investigation of context. Case study is one appropriate methodology, but there is little guidance about what case study design can offer the study of context in trials. We address this gap in the literature by presenting a number of important considerations for process evaluation using a case study design.

**Main text:**

In this paper, we define context, the relationship between complex interventions and context, and describe case study design methodology. A well-designed process evaluation using case study should consider the following core components: the purpose; definition of the intervention; the trial design, the case, the theories or logic models underpinning the intervention, the sampling approach and the conceptual or theoretical framework. We describe each of these in detail and highlight with examples from recently published process evaluations.

**Conclusions:**

There are a number of approaches to process evaluation design in the literature; however, there is a paucity of research on what case study design can offer process evaluations. We argue that case study is one of the best research designs to underpin process evaluations, to capture the dynamic and complex relationship between intervention and context during implementation. We provide a comprehensive overview of the issues for process evaluation design to consider when using a case study design.

**Trial registration:**

DQIP - ClinicalTrials.gov number, NCT01425502 - OPAL - ISRCTN57746448

Contribution to the literature

- We illustrate how case study methodology can explore the complex, dynamic and uncertain relationship between context and interventions within trials.
- We depict different case study designs and illustrate there is not one formula and that design needs to be tailored to the context and trial design.
- Case study can support comparisons between intervention and control arms and between cases within arms to uncover and explain differences in detail.
- We argue that case study can illustrate how components have evolved and been redefined through implementation.
- Key issues for consideration in case study design within process evaluations are presented and illustrated with examples.

## Background

Process evaluations are an important component of an effectiveness evaluation as they focus on understanding the relationship between interventions and context to explain how and why interventions work or fail and whether they can be transferred to other settings and populations. However, historically, not all trials have had a process evaluation component, nor have they sufficiently reported aspects of context, resulting in poor uptake of trial findings [1]. Considerations of context are often absent from published process evaluations, with few studies acknowledging, taking account of or describing context during implementation, or assessing the impact of context on implementation [2, 3]. At present, evidence from trials is not being used in a timely manner [4, 5], and this can negatively impact on patient benefit and experience [6]. It takes on average 17 years for knowledge from research to be implemented into practice [7]. Suitable methodologies are therefore needed that allow for context to be exposed; one appropriate methodological approach is case study [8, 9].

In 2015, the Medical Research Council (MRC) published guidance for process evaluations [10]. This was a key milestone in legitimising as well as providing tools, methods and a framework for conducting process evaluations. Nevertheless, as with all guidance, there is a need for reflection, challenge and refinement. There have been a number of critiques of the MRC guidance, including that interventions should be considered as events in systems [11–14]; a need for better use, critique and development of theories [15–17]; and a need for more guidance on integrating qualitative and quantitative data [18, 19]. Although the MRC process evaluation guidance does consider appropriate qualitative and quantitative methods, it does not mention case study design and what it can offer the study of context in trials.

The case study methodology is ideally suited to real-world, sustainable intervention development and evaluation because it can explore and examine contemporary complex phenomena, in depth, in numerous contexts and using multiple sources of data [8]. Case study design can capture the complexity of the case, the relationship between the intervention and the context and how the intervention worked (or not) [8]. There are a number of textbooks on a case study within the social science fields [8, 9, 20], but there are no case study textbooks and a paucity of useful texts on how to design, conduct and report case study within the health arena. Few examples exist within the trial design and evaluation literature [3, 21]. Therefore, guidance to enable well-designed process evaluations using case study methodology is required.

We aim to address the gap in the literature by presenting a number of important considerations for process evaluation using a case study design. First, we define the context and describe the relationship between complex health interventions and context.

### What is context?

While there is growing recognition that context interacts with the intervention to impact on the intervention’s effectiveness [22], context is still poorly defined and conceptualised. There are a number of different definitions in the literature, but as Bate et al. explained ‘almost universally, we find context to be an overworked word in everyday dialogue but a massively understudied and misunderstood concept’ [23]. Ovretveit defines context as ‘everything the intervention is not’ [24]. This last definition is used by the MRC framework for process evaluations [25]; however; the problem with this definition is that it is highly dependent on how the intervention is defined. We have found Pfadenhauer et al.’s definition useful:

Context is conceptualised as a set of characteristics and circumstances that consist of active and unique factors that surround the implementation. As such it is not a backdrop for implementation but interacts, influences, modifies and facilitates or constrains the intervention and its implementation. Context is usually considered in relation to an intervention or object, with which it actively interacts. A boundary between the concepts of context and setting is discernible: setting refers to the physical, specific location in which the intervention is put into practice. Context is much more versatile, embracing not only the setting but also roles, interactions and relationships [22].

Traditionally, context has been conceptualised in terms of barriers and facilitators, but what is a barrier in one context may be a facilitator in another, so it is the relationship and dynamics between the intervention and context which are the most important [26]. There is a need for empirical research to really understand how different contextual factors relate to each other and to the intervention. At present, research studies often list common contextual factors, but without a depth of meaning and understanding, such as government or health board policies, organisational structures, professional and patient attitudes, behaviours and beliefs [27]. The case study methodology is well placed to understand the relationship between context and intervention where these boundaries may not be clearly evident. It offers a means of unpicking the contextual conditions which are pertinent to effective implementation.

### The relationship between complex health interventions and context

Health interventions are generally made up of a number of different components and are considered complex due to the influence of context on their implementation and outcomes [3, 28]. Complex interventions are often reliant on the engagement of practitioners and patients, so their attitudes, behaviours, beliefs and cultures influence whether and how an intervention is effective or not. Interventions are context-sensitive; they interact with the environment in which they are implemented. In fact, many argue that interventions are a product of their context, and indeed, outcomes are likely to be a product of the intervention and its context [3, 29]. Within a trial, there is also the influence of the research context too—so the observed outcome could be due to the intervention alone, elements of the context within which the intervention is being delivered, elements of the research process or a combination of all three. Therefore, it can be difficult and unhelpful to separate the intervention from the context within which it was evaluated because the intervention and context are likely to have evolved together over time. As a result, the same intervention can look and behave differently in different contexts, so it is important this is known, understood and reported [3]. Finally, the intervention context is dynamic; the people, organisations and systems change over time, [3] which requires practitioners and patients to respond, and they may do this by adapting the intervention or contextual factors. So, to enable researchers to replicate successful interventions, or to explain why the intervention was not successful, it is not enough to describe the components of the intervention, they need to be described by their relationship to their context and resources [3, 28].

### What is a case study?

Case study methodology aims to provide an in-depth, holistic, balanced, detailed and complete picture of complex contemporary phenomena in its natural context [8, 9, 20]. In this case, the phenomena are the implementation of complex interventions in a trial. Case study methodology takes the view that the phenomena can be more than the sum of their parts and have to be understood as a whole [30]. It is differentiated from a clinical case study by its analytical focus [20].

The methodology is particularly useful when linked to trials because some of the features of the design naturally fill the gaps in knowledge generated by trials. Given the methodological focus on understanding phenomena in the round, case study methodology is typified by the use of multiple sources of data, which are more commonly qualitatively guided [31]. The case study methodology is not epistemologically specific, like realist evaluation, and can be used with different epistemologies [32], and with different theories, such as Normalisation Process Theory (which explores how staff work together to implement a new intervention) or the Consolidated Framework for Implementation Research (which provides a menu of constructs associated with effective implementation) [33–35]. Realist evaluation can be used to explore the relationship between context, mechanism and outcome, but case study differs from realist evaluation by its focus on a holistic and in-depth understanding of the relationship between an intervention and the contemporary context in which it was implemented [36]. Case study enables researchers to choose epistemologies and theories which suit the nature of the enquiry and their theoretical preferences.

### Designing a process evaluation using case study

An important part of any study is the research design. Due to their varied philosophical positions, the seminal authors in the field of case study have different epistemic views as to how a case study should be conducted [8, 9]. Stake takes an interpretative approach (interested in how people make sense of their world), and Yin has more positivistic leanings, arguing for objectivity, validity and generalisability [8, 9].

Regardless of the philosophical background, a well-designed process evaluation using case study should consider the following core components: the purpose; the definition of the intervention, the trial design, the case, and the theories or logic models underpinning the intervention; the sampling approach; and the conceptual or theoretical framework [8, 9, 20, 31, 33]. We now discuss these critical components in turn, with reference to two process evaluations that used case study design, the DQIP and OPAL studies [21, 37–41].

### Purpose

The purpose of a process evaluation is to evaluate and explain the relationship between the intervention and its components, to context and outcome. It can help inform judgements about validity (by exploring the intervention components and their relationship with one another (construct validity), the connections between intervention and outcomes (internal validity) and the relationship between intervention and context (external validity)). It can also distinguish between implementation failure (where the intervention is poorly delivered) and intervention failure (intervention design is flawed) [42, 43]. By using a case study to explicitly understand the relationship between context and the intervention during implementation, the process evaluation can explain the intervention effects and the potential generalisability and optimisation into routine practice [44].

The DQIP process evaluation aimed to qualitatively explore how patients and GP practices responded to an intervention designed to reduce high-risk prescribing of nonsteroidal anti-inflammatory drugs (NSAIDs) and/or antiplatelet agents (see Table 1) and quantitatively examine how change in high-risk prescribing was associated with practice characteristics and implementation processes. The OPAL process evaluation (see Table 2) aimed to quantitatively understand the factors which influenced the effectiveness of a pelvic floor muscle training intervention for women with urinary incontinence and qualitatively explore the participants’ experiences of treatment and adherence.

**Table 1:** Data-driven Quality Improvement in Primary Care (DQIP)

**Trial:** The DQIP trial was a cluster randomised, stepped wedge trial in 33 practices from one Scottish health board (NHS Tayside) which aimed to reduce high-risk prescribing of nonsteroidal anti-inflammatory drugs (NSAIDs) and/or selected antiplatelet agents. All practices received the intervention but were randomised to one of 10 different start dates. **Intervention:** The DQIP intervention comprised education, an informatics tool and a financial incentive. In DQIP, the researchers delivered education and training to the general practices (clusters) with high fidelity (of form and function); the general practices were then free to organise themselves as they saw fit to deliver the intervention to patients (fidelity of function, but variation in form). For the process evaluation design, the DQIP intervention was conceptualised as two interventions; the intervention delivered to clusters (intervention 1), and the intervention delivered to patients (intervention 2) as there may be different processes working at the cluster and individual patient level. Data were collected on intervention 1 in all practices and in a purposive sample for intervention 2, using a comparative case study design for both interventions. **Purpose**: The DQIP mixed method process evaluation aimed to qualitatively explore how patients and practices responded to the intervention and quantitatively examine how a change in high risk prescribing was associated with practice characteristics and implementation processes. **Qualitative design**: A mixed method multiple comparative case study with general practices as the units of analysis. **Quantitative design**: Prespecified analysis to explore associations between practice characteristics, implementation processes and change in prescribing. **Sample**: One practice was sampled per cohort of the trial. Ten general practices were purposively sampled based on their initial adoption of the intervention, four practices which rapidly implemented the intervention and six who were initial implementation failures. **Data collection**: Routine data collected during the trial were used to inform the quantitative part of the process evaluation, and to mirror the trial design, a case was selected for qualitative data collection from each cohort in the trial. **Conceptual and theoretical framework:** Framework for process evaluation design [45] and Normalisation Process Theory [34]. **Analysis:** An in-depth description of each case was constructed detailing the practice characteristics and perceptions of all staff who participated in the interviews, with additional data from the educational outreach observation and informal interviews. Deductive theoretical analysis using the Normalisation Process Theory was also conducted. The Framework technique of matrixes facilitated detailed exploration by theoretical construct theme, practice and cross- and within-case comparisons*.* Thematic and theoretical saturation was achieved. **What did the case study process evaluation design reveal about context?** Case study design illustrated that to achieve effective implementation agreement that the topic (NSAIDs and antiplatelets) was important among all clinical staff was fundamental. In addition, that practices made plans early in the process to implement the intervention including responsibility for the work and regularly evaluated their progress. Also, how practices internally organised to do the work varied illustrating this was not important for effective evaluation. Case study design was important for illustrating that implementation failure occurred at different stages depending on the practice culture and context, illuminating the differences in organisational processes and the contextual and organisational factors which impacted on effective implementation. The case study’s holistic approach to understanding how the context and culture within each practice influenced processes was key to explaining whether the intervention worked or not. Also, practice context was not fixed, so most practices adapted and were able to deliver some elements. **DQIP case study design model**

**Table 2:** Optimising Pelvic Floor Exercises to Achieve Long-term benefits (OPAL)

**Trial:** The OPAL trial was a large multi-centre pragmatic randomised controlled trial of two active treatment arms delivered across 23 primary and secondary care sites for 600 women. **Intervention:** OPAL aimed to determine the effectiveness of two active treatment arms: basic pelvic floor muscle training (PFMT) and basic pelvic floor muscle training (PFMT) with biofeedback mediated intensive for the treatment of stress or mixed (stress and urgency) female urinary incontinence. **Purpose:** The OPAL trial has an embedded mixed methods process evaluation and a longitudinal qualitative case study, which aim to explain the trial outcomes. The longitudinal qualitative study aimed to investigate women’s experiences and adherence to the interventions. **Qualitative design:** A two-tailed embedded multiple case study design utilising longitudinal interviews. Within a multiple case study design, Yin outlines a ‘two-tailed’ approach where cases are selected to represent two extremities in relation to phenomena; in this case, the extremities are the control and intervention groups [46]. Units of analysis were at the individual case (the participants) and the trial arms (intervention and control). There were two units of analysis to enable an in-depth exploration within each case but also at the trial arm level to explore commonalities and differences between the cases in each arm and between the arms. **Quantitative design**: Prespecified analysis to explore fidelity, engagement and mediating factors using descriptive and interpretative statistics. **Sample:** The two-tailed case study design means multiple cases (*n* = 20) from each trial arm were sampled to enable comparison between the trial arms. Cases were purposively sampled for variance in centre type (university hospital, district general hospital or community delivered service), therapist delivery type (physiotherapist/nurse), women’s type of UI (stress or mixed) and over time to reflect recruitment to the trial. **Data collection:** Mirroring the trial data collection, the case study was longitudinal, with women interviewed four times (baseline, post-treatment, 12 months and 24 months post-randomisation). Twenty women per arm were recruited, and 24 women across both arms were interviewed 4 times. GF **Conceptual framework:** Framework for process evaluation design [45]. **Analysis:** A case was built and summarised over 2 years, with four data points for each woman. Case summaries were written summarising women’s experiences. Theoretical propositions were developed to guide the analysis. All the cases for one trial arm were grouped and within arm consistencies/inconsistencies searched for. The experimental and comparator tails were compared to one another using the theoretical propositions. Thematic and theoretical saturation was achieved. **What did the case study process evaluation design reveal about context?** Adherence to the interventions in the OPAL trial was hugely variable; in both trial arms, there were some women who had good adherence, some who were adherent at certain time points and some who did not adhere well at all. The case study was useful in illuminating the ways in which the context of the participants’ lives influenced adherence in both trial arms. The temporal nature of the data collection and case studies was useful in illustrating that the context of the women’s lives was dynamic, and thus, their engagement with the interventions was also dynamic. The in-depth case studies were useful in illustrating that based on each participants’ unique circumstances, different contextual factors and personal characteristics were important, such as their motivation to maintain engagement. Across-arm case comparison was able to illustrate that these factors were not related to the interventions but specific to the participants. Across-arm comparisons showed that although many participants had not maintained adherence, they felt more skilled in pelvic floor muscle training (PFMT) and able to restart PFMT exercise after a break [47, 48].	

### Defining the intervention and exploring the theories or assumptions underpinning the intervention design

Process evaluations should also explore the utility of the theories or assumptions underpinning intervention design [49]. Not all theories underpinning interventions are based on a formal theory, but they based on assumptions as to how the intervention is expected to work. These can be depicted as a logic model or theory of change [25]. To capture how the intervention and context evolve requires the intervention and its expected mechanisms to be clearly defined at the outset [50]. Hawe and colleagues recommend defining interventions by function (what processes make the intervention work) rather than form (what is delivered) [51]. However, in some cases, it may be useful to know if some of the components are redundant in certain contexts or if there is a synergistic effect between all the intervention components.

The DQIP trial delivered two interventions, one intervention was delivered to professionals with high fidelity and then professionals delivered the other intervention to patients by form rather than function allowing adaptations to the local context as appropriate. The assumptions underpinning intervention delivery were prespecified in a logic model published in the process evaluation protocol [52].

Case study is well placed to challenge or reinforce the theoretical assumptions or redefine these based on the relationship between the intervention and context. Yin advocates the use of theoretical propositions; these direct attention to specific aspects of the study for investigation [8] can be based on the underlying assumptions and tested during the course of the process evaluation. In case studies, using an epistemic position more aligned with Yin can enable research questions to be designed, which seek to expose patterns of unanticipated as well as expected relationships [9]. The OPAL trial was more closely aligned with Yin, where the research team predefined some of their theoretical assumptions, based on how the intervention was expected to work. The relevant parts of the data analysis then drew on data to support or refute the theoretical propositions. This was particularly useful for the trial as the prespecified theoretical propositions linked to the mechanisms of action on which the intervention was anticipated to have an effect (or not).

### Tailoring to the trial design

Process evaluations need to be tailored to the trial, the intervention and the outcomes being measured [45]. For example, in a stepped wedge design (where the intervention is delivered in a phased manner), researchers should try to ensure process data are captured at relevant time points or in a two-arm or multiple arm trial, ensure data is collected from the control group(s) as well as the intervention group(s). In the DQIP trial, a stepped wedge trial, at least one process evaluation case, was sampled per cohort. Trials often continue to measure outcomes after delivery of the intervention has ceased, so researchers should also consider capturing ‘follow-up’ data on contextual factors, which may continue to influence the outcome measure. The OPAL trial had two active treatment arms so collected process data from both arms. In addition, as the trial was interested in long-term adherence, the trial and the process evaluation collected data from participants for 2 years after the intervention was initially delivered, providing 24 months follow-up data, in line with the primary outcome for the trial.

### Defining the case

Case studies can include single or multiple cases in their design. Single case studies usually sample typical or unique cases, their advantage being the depth and richness that can be achieved over a long period of time. The advantages of multiple case study design are that cases can be compared to generate a greater depth of analysis. Multiple case study sampling may be carried out in order to test for replication or contradiction [8]. Given that trials are often conducted over a number of sites, a multiple case study design is more sensible for process evaluations, as there is likely to be variation in implementation between sites. Case definition may occur at a variety of levels but is most appropriate if it reflects the trial design. For example, a case in an individual patient level trial is likely to be defined as a person/patient (e.g. a woman with urinary incontinence—OPAL trial) whereas in a cluster trial, a case is like to be a cluster, such as an organisation (e.g. a general practice—DQIP trial). Of course, the process evaluation could explore cases with less distinct boundaries, such as communities or relationships; however, the clarity with which these cases are defined is important, in order to scope the nature of the data that will be generated.

### Sampling

Carefully sampled cases are critical to a good case study as sampling helps inform the quality of the inferences that can be made from the data [53]. In both qualitative and quantitative research, how and how many participants to sample must be decided when planning the study. Quantitative sampling techniques generally aim to achieve a random sample. Qualitative research generally uses purposive samples to achieve data saturation, occurring when the incoming data produces little or no new information to address the research questions. The term data saturation has evolved from theoretical saturation in conventional grounded theory studies; however, its relevance to other types of studies is contentious as the term saturation seems to be widely used but poorly justified [54]. Empirical evidence suggests that for in-depth interview studies, saturation occurs at 12 interviews for thematic saturation, but typically more would be needed for a heterogenous sample higher degrees of saturation [55, 56]. Both DQIP and OPAL case studies were huge with OPAL designed to interview each of the 40 individual cases four times and DQIP designed to interview the lead DQIP general practitioner (GP) twice (to capture change over time), another GP and the practice manager from each of the 10 organisational cases. Despite the plethora of mixed methods research textbooks, there is very little about sampling as discussions typically link to method (e.g. interviews) rather than paradigm (e.g. case study).

Purposive sampling can improve the generalisability of the process evaluation by sampling for greater contextual diversity. The typical or average case is often not the richest source of information. Outliers can often reveal more important insights, because they may reflect the implementation of the intervention using different processes. Cases can be selected from a number of criteria, which are not mutually exclusive, to enable a rich and detailed picture to be built across sites [53]. To avoid the Hawthorne effect, it is recommended that process evaluations sample from both intervention and control sites, which enables comparison and explanation. There is always a trade-off between breadth and depth in sampling, so it is important to note that often quantity does not mean quality and that carefully sampled cases can provide powerful illustrative examples of how the intervention worked in practice, the relationship between the intervention and context and how and why they evolved together. The qualitative components of both DQIP and OPAL process evaluations aimed for maximum variation sampling. Please see Table 1 for further information on how DQIP’s sampling frame was important for providing contextual information on processes influencing effective implementation of the intervention.

### Conceptual and theoretical framework

A conceptual or theoretical framework helps to frame data collection and analysis [57]. Theories can also underpin propositions, which can be tested in the process evaluation. Process evaluations produce intervention-dependent knowledge, and theories help make the research findings more generalizable by providing a common language [16]. There are a number of mid-range theories which have been designed to be used with process evaluation [34, 35, 58]. The choice of the appropriate conceptual or theoretical framework is, however, dependent on the philosophical and professional background of the research. The two examples within this paper used our own framework for the design of process evaluations, which proposes a number of candidate processes which can be explored, for example, recruitment, delivery, response, maintenance and context [45]. This framework was published before the MRC guidance on process evaluations, and both the DQIP and OPAL process evaluations were designed before the MRC guidance was published. The DQIP process evaluation explored all candidates in the framework whereas the OPAL process evaluation selected four candidates, illustrating that process evaluations can be selective in what they explore based on the purpose, research questions and resources. Furthermore, as Kislov and colleagues argue, we also have a responsibility to critique the theoretical framework underpinning the evaluation and refine theories to advance knowledge [59].

### Data collection

An important consideration is what data to collect or measure and when. Case study methodology supports a range of data collection methods, both qualitative and quantitative, to best answer the research questions. As the aim of the case study is to gain an in-depth understanding of phenomena in context, methods are more commonly qualitative or mixed method in nature. Qualitative methods such as interviews, focus groups and observation offer rich descriptions of the setting, delivery of the intervention in each site and arm, how the intervention was perceived by the professionals delivering the intervention and the patients receiving the intervention. Quantitative methods can measure recruitment, fidelity and dose and establish which characteristics are associated with adoption, delivery and effectiveness. To ensure an understanding of the complexity of the relationship between the intervention and context, the case study should rely on multiple sources of data and triangulate these to confirm and corroborate the findings [8]. Process evaluations might consider using routine data collected in the trial across all sites and additional qualitative data across carefully sampled sites for a more nuanced picture within reasonable resource constraints. Mixed methods allow researchers to ask more complex questions and collect richer data than can be collected by one method alone [60]. The use of multiple sources of data allows data triangulation, which increases a study’s internal validity but also provides a more in-depth and holistic depiction of the case [20]. For example, in the DQIP process evaluation, the quantitative component used routinely collected data from all sites participating in the trial and purposively sampled cases for a more in-depth qualitative exploration [21, 38, 39].

The timing of data collection is crucial to study design, especially within a process evaluation where data collection can potentially influence the trial outcome. Process evaluations are generally in parallel or retrospective to the trial. The advantage of a retrospective design is that the evaluation itself is less likely to influence the trial outcome. However, the disadvantages include recall bias, lack of sensitivity to nuances and an inability to iteratively explore the relationship between intervention and outcome as it develops. To capture the dynamic relationship between intervention and context, the process evaluation needs to be parallel and longitudinal to the trial. Longitudinal methodological design is rare, but it is needed to capture the dynamic nature of implementation [40]. How the intervention is delivered is likely to change over time as it interacts with context. For example, as professionals deliver the intervention, they become more familiar with it, and it becomes more embedded into systems. The OPAL process evaluation was a longitudinal, mixed methods process evaluation where the quantitative component had been predefined and built into trial data collection systems. Data collection in both the qualitative and quantitative components mirrored the trial data collection points, which were longitudinal to capture adherence and contextual changes over time.

There is a lot of attention in the recent literature towards a systems approach to understanding interventions in context, which suggests interventions are ‘events within systems’ [61, 62]. This framing highlights the dynamic nature of context, suggesting that interventions are an attempt to change systems dynamics. This conceptualisation would suggest that the study design should collect contextual data before and after implementation to assess the effect of the intervention on the context and vice versa.

### Data analysis

Designing a rigorous analysis plan is particularly important for multiple case studies, where researchers must decide whether their approach to analysis is case or variable based. Case-based analysis is the most common, and analytic strategies must be clearly articulated for within and across case analysis. A multiple case study design can consist of multiple cases, where each case is analysed at the case level, or of multiple embedded cases, where data from all the cases are pulled together for analysis at some level. For example, OPAL analysis was at the case level, but all the cases for the intervention and control arms were pulled together at the arm level for more in-depth analysis and comparison. For Yin, analytical strategies rely on theoretical propositions, but for Stake, analysis works from the data to develop theory. In OPAL and DQIP, case summaries were written to summarise the cases and detail within-case analysis. Each of the studies structured these differently based on the phenomena of interest and the analytic technique. DQIP applied an approach more akin to Stake [9], with the cases summarised around inductive themes whereas OPAL applied a Yin [8] type approach using theoretical propositions around which the case summaries were structured. As the data for each case had been collected through longitudinal interviews, the case summaries were able to capture changes over time. It is beyond the scope of this paper to discuss different analytic techniques; however, to ensure the holistic examination of the intervention(s) in context, it is important to clearly articulate and demonstrate how data is integrated and synthesised [31].

## Conclusion

There are a number of approaches to process evaluation design in the literature; however, there is a paucity of research on what case study design can offer process evaluations. We argue that case study is one of the best research designs to underpin process evaluations, to capture the dynamic and complex relationship between intervention and context during implementation [38]. Case study can enable comparisons within and across intervention and control arms and enable the evolving relationship between intervention and context to be captured holistically rather than considering processes in isolation. Utilising a longitudinal design can enable the dynamic relationship between context and intervention to be captured in real time. This information is fundamental to holistically explaining what intervention was implemented, understanding how and why the intervention worked or not and informing the transferability of the intervention into routine clinical practice.

Case study designs are not prescriptive, but process evaluations using case study should consider the purpose, trial design, the theories or assumptions underpinning the intervention, and the conceptual and theoretical frameworks informing the evaluation. We have discussed each of these considerations in turn, providing a comprehensive overview of issues for process evaluations using a case study design. There is no single or best way to conduct a process evaluation or a case study, but researchers need to make informed choices about the process evaluation design. Although this paper focuses on process evaluations, we recognise that case study design could also be useful during intervention development and feasibility trials. Elements of this paper are also applicable to other study designs involving trials.

## Data Availability

No data and materials were used.

## Abbreviations

DQIP: Data-driven Quality Improvement in Primary Care
MRC: Medical Research Council
NSAIDs: Nonsteroidal anti-inflammatory drugs
OPAL: Optimizing Pelvic Floor Muscle Exercises to Achieve Long-term benefits

## References

[1] Blencowe NB (2015). Systematic review of intervention design and delivery in pragmatic and explanatory surgical randomized clinical trials. Br J Surg.

[2] Dixon-Woods M. The problem of context in quality improvement. In: Foundation TH, editor. Perspectives on context: The Health Foundation; 2014.

[3] Wells M, Williams B, Treweek S, Coyle J, Taylor J (2012). Intervention description is not enough: evidence from an in-depth multiple case study on the untold role and impact of context in randomised controlled trials of seven complex interventions. Trials.

[4] Grant A, Sullivan F, Dowell J (2013). An ethnographic exploration of influences on prescribing in general practice: why is there variation in prescribing practices?. Implement Sci.

[5] Lang ES, Wyer PC, Haynes RB (2007). Knowledge translation: closing the evidence-to-practice gap. Ann Emerg Med.

[6] Ward V, House AF, Hamer S (2009). Developing a framework for transferring knowledge into action: a thematic analysis of the literature. J Health Serv Res Policy.

[7] Morris ZS, Wooding S, Grant J (2011). The answer is 17 years, what is the question: understanding time lags in translational research. J R Soc Med.

[8] Yin R (2018). Case study research and applications: design and methods.

[9] Stake R (1995). The art of case study research.

[10] Moore GF, Audrey S, Barker M, Bond L, Bonell C, Hardeman W, Moore L, O’Cathain A, Tinati T, Wight D, et al. Process evaluation of complex interventions: Medical Research Council guidance. Br Med J. 2015;350. 10.1136/bmj.h1258 PMC4366184 25791983

[11] Hawe P (2015). Minimal, negligible and negligent interventions. Soc Sci Med.

[12] Moore GF, Evans RE, Hawkins J, Littlecott H, Melendez-Torres GJ, Bonell C, Murphy S (2018). From complex social interventions to interventions in complex social systems: future directions and unresolved questions for intervention development and evaluation. Evaluation.

[13] Greenhalgh T, Papoutsi C (2018). Studying complexity in health services research: desperately seeking an overdue paradigm shift. BMC Med.

[14] Rutter H, Savona N, Glonti K, Bibby J, Cummins S, Finegood DT, Greaves F, Harper L, Hawe P, Moore L (2017). The need for a complex systems model of evidence for public health. Lancet.

[15] Moore G, Cambon L, Michie S, Arwidson P, Ninot G, Ferron C, Potvin L, Kellou N, Charlesworth J, Alla F (2019). Population health intervention research: the place of theories. Trials.

[16] Kislov R (2019). Engaging with theory: from theoretically informed to theoretically informative improvement research. BMJ Qual Saf.

[17] Boulton R, Sandall J, Sevdalis N. The cultural politics of ‘Implementation Science’. J Med Human. 2020;41(3):379-94. h10.1007/s10912-020-09607-9. 10.1007/s10912-020-09607-9 PMC7343725 31965463

[18] Cheng KKF, Metcalfe A (2018). Qualitative methods and process evaluation in clinical trials context: where to head to?. Int J Qual Methods.

[19] Richards DA, Bazeley P, Borglin G, Craig P, Emsley R, Frost J, Hill J, Horwood J, Hutchings HA, Jinks C (2019). Integrating quantitative and qualitative data and findings when undertaking randomised controlled trials. BMJ Open.

[20] Thomas G (2016). How to do your case study, 2nd edition edn.

[21] Grant A, Dreischulte T, Guthrie B. Process evaluation of the Data-driven Quality Improvement in Primary Care (DQIP) trial: case study evaluation of adoption and maintenance of a complex intervention to reduce high-risk primary care prescribing. BMJ Open. 2017;7(3). 10.1136/bmjopen-2016-015281 PMC5353272 28283493

[22] Pfadenhauer L, Rohwer A, Burns J, Booth A, Lysdahl KB, Hofmann B, Gerhardus A, Mozygemba K, Tummers M, Wahlster P, et al. Guidance for the assessment of context and implementation in health technology assessments (HTA) and systematic reviews of complex interventions: the Context and Implementation of Complex Interventions (CICI) framework: Integrate-HTA; 2016.

[23] Bate P, Robert G, Fulop N, Ovretveit J, Dixon-Woods M (2014). Perspectives on context.

[24] Ovretveit J. Understanding the conditions for improvement: research to discover which context influences affect improvement success. BMJ Qual Saf. 2011;20. 10.1136/bmjqs.2010.045955 PMC3066695 21450764

[25] Medical Research Council (2015). Process evaluation of complex interventions: UK Medical Research Council (MRC) guidance.

[26] May CR, Johnson M, Finch T (2016). Implementation, context and complexity. Implement Sci.

[27] Bate P. Context is everything. In: Perpesctives on Context. The Health Foundation 2014.

[28] Horton TJ, Illingworth JH, Warburton WHP (2018). Overcoming challenges in codifying and replicating complex health care interventions. Health Aff.

[29] O'Connor AM, Tugwell P, Wells GA, Elmslie T, Jolly E, Hollingworth G, McPherson R, Bunn H, Graham I, Drake E (1998). A decision aid for women considering hormone therapy after menopause: decision support framework and evaluation. Patient Educ Couns.

[30] Creswell J, Poth C (2018). Qualiative inquiry and research design, fourth edition edn.

[31] Carolan CM, Forbat L, Smith A (2016). Developing the DESCARTE model: the design of case study research in health care. Qual Health Res.

[32] Takahashi ARW, Araujo L (2020). Case study research: opening up research opportunities. RAUSP Manage J.

[33] Tight M (2017). Understanding case study research, small-scale research with meaning.

[34] May C, Finch T (2009). Implementing, embedding, and integrating practices: an outline of normalisation process theory. Sociology.

[35] Damschroder LJ, Aron DC, Keith RE, Kirsh SR, Alexander JA, Lowery JC. Fostering implementation of health services research findings into practice. A consolidated framework for advancing implementation science. Implement Sci. 2009;4. 10.1186/1748-5908-4-50 PMC2736161 19664226

[36] Pawson R, Tilley N (1997). Realist evaluation.

[37] Dreischulte T, Donnan P, Grant A, Hapca A, McCowan C, Guthrie B (2016). Safer prescribing - a trial of education, informatics & financial incentives. N Engl J Med.

[38] Grant A, Dreischulte T, Guthrie B (2017). Process evaluation of the Data-driven Quality Improvement in Primary Care (DQIP) trial: active and less active ingredients of a multi-component complex intervention to reduce high-risk primary care prescribing. Implement Sci.

[39] Dreischulte T, Grant A, Hapca A, Guthrie B (2018). Process evaluation of the Data-driven Quality Improvement in Primary Care (DQIP) trial: quantitative examination of variation between practices in recruitment, implementation and effectiveness. BMJ Open.

[40] Grant A, Dean S, Hay-Smith J, Hagen S, McClurg D, Taylor A, Kovandzic M, Bugge C (2019). Effectiveness and cost-effectiveness randomised controlled trial of basic versus biofeedback-mediated intensive pelvic floor muscle training for female stress or mixed urinary incontinence: protocol for the OPAL (Optimising Pelvic Floor Exercises to Achieve Long-term benefits) trial mixed methods longitudinal qualitative case study and process evaluation. BMJ Open.

[41] Hagen S, McClurg D, Bugge C, Hay-Smith J, Dean SG, Elders A, Glazener C, Abdel-fattah M, Agur WI, Booth J (2019). Effectiveness and cost-effectiveness of basic versus biofeedback-mediated intensive pelvic floor muscle training for female stress or mixed urinary incontinence: protocol for the OPAL randomised trial. BMJ Open.

[42] Steckler A, Linnan L (2002). Process evaluation for public health interventions and research.

[43] Durlak JA (1998). Why programme implementation is so important. J Prev Intervent Commun.

[44] Bonell C, Oakley A, Hargreaves J, VS, Rees R (2006). Assessment of generalisability in trials of health interventions: suggested framework and systematic review. Br Med J.

[45] Grant A, Treweek S, Dreischulte T, Foy R, Guthrie B (2013). Process evaluations for cluster-randomised trials of complex interventions: a proposed framework for design and reporting. Trials.

[46] Yin R (2003). Case study research: design and methods.

[47] Bugge C, Hay-Smith J, Grant A, Taylor A, Hagen S, McClurg D, Dean S: A 24 month longitudinal qualitative study of women’s experience of electromyography biofeedback pelvic floor muscle training (PFMT) and PFMT alone for urinary incontinence: adherence, outcome and context. ICS Gothenburg 2019 2019. https://www.ics.org/2019/abstract/473. Access 10.9.2020.

[48] Suzanne Hagen, Andrew Elders, Susan Stratton, Nicole Sergenson, Carol Bugge, Sarah Dean, Jean Hay-Smith, Mary Kilonzo, Maria Dimitrova, Mohamed Abdel-Fattah, Wael Agur, Jo Booth, Cathryn Glazener, Karen Guerrero, Alison McDonald, John Norrie, Louise R Williams, Doreen McClurg. Effectiveness of pelvic floor muscle training with and without electromyographic biofeedback for urinary incontinence in women: multicentre randomised controlled trial BMJ 2020;371. 10.1136/bmj.m3719. 10.1136/bmj.m3719 PMC7555069 33055247

[49] Cook TD (2005). Emergent principles for the design, implementation, and analysis of cluster-based experiments in social science. Ann Am Acad Pol Soc Sci.

[50] Hoffmann T, Glasziou P, Boutron I, Milne R, Perera R, Moher D. Better reporting of interventions: template for intervention description and replication (TIDieR) checklist and guide. Br Med J. 2014;348. 10.1136/bmj.g1687 24609605

[51] Hawe P, Shiell A, Riley T (2004). Complex interventions: how “out of control” can a randomised controlled trial be?. Br Med J.

[52] Grant A, Dreischulte T, Treweek S, Guthrie B (2012). Study protocol of a mixed-methods evaluation of a cluster randomised trial to improve the safety of NSAID and antiplatelet prescribing: Data-driven Quality Improvement in Primary Care. Trials.

[53] Flyvbjerg B (2006). Five misunderstandings about case-study research. Qual Inq.

[54] Thorne S (2020). The great saturation debate: what the “S word” means and doesn’t mean in qualitative research reporting. Can J Nurs Res.

[55] Guest G, Bunce A, Johnson L (2006). How many interviews are enough?: an experiment with data saturation and variability. Field Methods.

[56] Guest G, Namey E, Chen M (2020). A simple method to assess and report thematic saturation in qualitative research. PLoS One.

[57] Davidoff F, Dixon-Woods M, Leviton L, Michie S (2015). Demystifying theory and its use in improvement. BMJ Qual Saf.

[58] Rycroft-Malone J. The PARIHS framework: a framework for guiding the implementation of evidence-based practice. J Nurs Care Qual. 2004;4:297-304. 10.1097/00001786-200410000-00002 15535533

[59] Kislov R, Pope C, Martin GP, Wilson PM (2019). Harnessing the power of theorising in implementation science. Implement Sci.

[60] Cresswell JW, Plano Clark VL (2007). Designing and conducting mixed methods research.

[61] Hawe P, Shiell A, Riley T (2009). Theorising interventions as events in systems. Am J Community Psychol.

[62] Craig P, Ruggiero E, Frohlich KL, Mykhalovskiy E, White M. Taking account of context in population health intervention research: guidance for producers, users and funders of research: National Institute for Health Research; 2018.https://www.ncbi.nlm.nih.gov/books/NBK498645/pdf/Bookshelf_NBK498645.pdf.

